# Association of mastocytosis with interleukin 31 gene polymorphism

**DOI:** 10.1186/2045-7022-5-S1-P11

**Published:** 2015-03-11

**Authors:** Boguslaw Nedoszytko, Monika Zabotna, Marek Niedoszytko, Magdalena Lange

**Affiliations:** 1 Medical University of Gdansk, Department of Dermatology, Gdansk; Germany; 2 Medical University of Gdansk, Department of Allergology, Gdansk, Germany

## 

Mastocytosis is a rare disease characterized by activating *KIT* mutations and clonal expansion of mast cells (MCs) in tissues, particularly in skin and bone marrow. Symptoms of mastocytosis always correlate with the extent of MC infiltration and include urticaria pigmentosa, anaphylaxis, flushing, headache, diarrhea, osteoporosis and pruritus. Interleukin 31 (IL-31) is a new recently identified pruritogenic factor mainly produced by activated T cells, but also by mast cells. Recently published data indicated that IL-31 level is increased in adult patients with mastocytosis.

The aim of our study is to compare the frequency of -1066 G/A, -2057 G/A and IVS2+12 polymorphisms of IL-31 gene which upregulate gene transcription in mastocytosis patients and healthy control groups.

Using ARMS-PCR methods we analyze the polymorphisms of IL-31 gene in 240 mastocytosis patients and 110 healthy controls.

We have found that in comparison to the control mastocytosis patients have more frequently AA genotype in all three analyzed polymorphisms: -1066 AA: 17,7% vs 8,2%, p=0,02; -2057 AA: 7,5% vs 1,1%, p=0,024, IVS2+12 AA 63,6 vs 40%, p=0,007. The presence of -2057 AA genotype more than 7x increase the risk of mastocytosis: OR = 7,38 (0,97-56.09) p = 0,05.

In conclusion, results of our study indicate that mastocytosis could be associated with IL-31 gene polymorphisms

